# Assessing error awareness without relying on introspective judgment?

**DOI:** 10.3389/fnins.2013.00113

**Published:** 2013-07-08

**Authors:** Tilmann A. Klein, Markus Ullsperger, Claudia Danielmeier

**Affiliations:** ^1^Department of Neurology, Max Planck Institute for Human Cognitive and Brain SciencesLeipzig, Germany; ^2^Day Clinic for Cognitive Neurology, University Clinic LeipzigLeipzig, Germany; ^3^Donders Institute for Brain, Cognition, and Behaviour, Radboud University NijmegenNijmegen, Netherlands; ^4^Institute of Psychology, Otto-von-Guericke University MagdeburgMagdeburg, Germany

Becoming aware of one's own mistake may be a prerequisite for remedial and compensatory actions. Improving error awareness is of great interest for situations of human life in which either a high accuracy is emphasized or in which people have to communicate errors among each other in order to improve group performance. Having a closer understanding of what makes a person aware of an error and which brain correlates are involved in this process might open new perspectives for enhancing error awareness in multiple fields of everyday life.

Research on error awareness has to deal with two fundamental issues: how to manipulate and how to measure conscious error perception.

What all error awareness studies have in common is that subjects are required to signal the commission of an error. Only with this subjective evaluation has it been possible to categorize errors into “aware” and “unaware.” Two different procedures have been used for this: either participants only press a dedicated “error signaling button,” when they feel that their last response has been wrong, or subjects are required to evaluate their response after each trial. Both procedures have disadvantages (Klein et al., 2013). Therefore, it would be advantageous, if subjective error awareness could be deduced from the subject's brain activity without asking participants directly. A recent study by Murphy and colleagues (2012) provides deeper insights for these considerations.

Two event-related potentials correlate with different aspects of error processing: the error-related negativity (ERN; Falkenstein et al., 1990; Gehring et al., 1993) and the error positivity (Pe, Falkenstein et al., 1990). The functional role of the ERN has been the subject of several elaborate theories (Falkenstein et al., 1991; Botvinick et al., 2001; Holroyd and Coles, 2002; Yeung et al., 2004). In contrast, the generation and the functional significance of the Pe seem less clear (Overbeek et al., 2005; Ridderinkhof et al., 2009). Theories of the role of the Pe range from being a representation of the motivational valence of the erroneous response (Leuthold and Sommer, 1999) to being a correlate of evidence accumulation of error occurrence [i.e., subject's error awareness (Steinhauser and Yeung, 2010; Ullsperger et al., 2010)].

Earlier electrophysiological research concluded that a measurable Pe is only detectable after a consciously perceived error (Nieuwenhuis et al., 2001; Endrass et al., 2005, 2007; for a discussion of the role of the ERN in error awareness: Scheffers and Coles, 2000; Wessel et al., 2011; Wessel, 2012). However, previous electrophysiological research (e.g., Nieuwenhuis et al., 2001; Endrass et al., 2005, 2007; see also Klein et al., 2007) on error awareness has always been performed with group-averaged data, and analyses were time-locked to the erroneous response thereby neglecting that processes related to the erroneous button press (and most likely related to the ERN) might be dissociable from processes underlying error-awareness and the Pe (Hughes and Yeung, 2011). Indeed, there could be a significant temporal jitter between the erroneous response and the onset of error awareness processes, which could be especially problematic in group studies comparing patients with healthy controls. If the timing between error commission and the onset of the Pe differs between groups, researchers might find spurious differences in Pe amplitudes between groups when they analyze the Pe time-locked to the erroneous button press. The study by Murphy et al. (2012) elegantly addressed these issues and thereby the functional significance of the Pe. The study provides the basis for more substantiated conclusions on the functionality of the Pe. By using an EEG variant of the Go/NoGo error awareness task (cf. Hester et al., 2005; O'Connell et al., 2007) they investigated the process of becoming aware of an error and its correlation to different aspects of the Pe. Participants indicated error awareness with a speeded button press thereby supplying the researchers with precise timing information about the awareness process. To get a pure single trial measure of the Pe and to have access to the within trial variability in amplitude and latency they used independent-component analysis (ICA, Makeig et al., 2004) in order to isolate a Pe-component that is free of any potential contaminations (muscle artifacts, stimulus processing etc.). They used these individually determined components to correlate them (within- and between-subject) with behavioral indices of error awareness: the reaction time and the frequency of the error signaling response. In contrast to earlier studies, where Pe analyses were time-locked to the erroneous response, Murphy and colleagues showed that the Pe amplitude and latency is more strongly related to the awareness button press than to the preceding erroneous response. Precise Pe analyses should therefore take the timing of the subjective awareness signaling into account to avoid the amplitude being affected by a signaling latency jitter. Using receiver-operating-characteristic (ROC) analysis the authors showed that the Pe could be used to predict an awareness response well before this response was issued. Murphy and colleagues furthermore demonstrated that Pe amplitude and latency are predictive for the proportion of awareness responses on the between subject level. The authors finally conclude that the Pe correlates with the cumulative development of error awareness.

The present paper is remarkable due to both its methodological improvements and the advancements in understanding the process of becoming aware of an error as well as the functional role of the Pe in this process. As discussed by the authors, direct improvements for future studies can be derived: the use of a speeded error-signaling response (see also Ullsperger and Von Cramon, 2006) seems useful to measure the precise timing of development of awareness on the individual level. Implications for clinical studies investigating patients with deficits in error awareness might be that observed differences in Pe amplitude might also be an “artifact” due to timing differences in the process of becoming aware of an error (and thereby latency differences in Pe). The authors therefore suggest investigating error awareness with respect to the error signaling response and not with respect to the erroneous response as such. The cumulative nature of the awareness process that the authors suggest fits well with the central role that is often assumed for the anterior insula in error awareness (see Klein et al., 2013 for a recent review).

Might it become possible to directly infer error awareness from the individual response of the subject's brain (i.e., the Pe) without an explicit awareness response? Murphy and colleagues showed on the one hand that ROC analyses on post-response brain data are indeed able to predict error awareness with high accuracy. On the other hand, they pointed out that error signaling responses are needed for more accurate Pe analyses. Especially for clinical studies comparing different groups of patients with healthy controls, a speeded subjective response evaluation could clearly improve research on error awareness processes. A better understanding of awareness processes and their neurophysiological correlates might also stimulate research on interventions that might help to promote error awareness and on factors that reduce error awareness (e.g., neurological diseases, psychiatric illness, drugs, tiredness, etc.).
